# Observations on an Aggregation of Grey Reef Sharks (*Carcharhinus amblyrhynchos*) in the Mozambique Channel Off the Coast of Nosy Be (Madagascar) and Tools for Photo-Identification—A New Aggregation Nursery Site?

**DOI:** 10.3390/biology13090661

**Published:** 2024-08-26

**Authors:** Primo Micarelli, Marco Pireddu, Damiano Persia, Marco Sanna, Consuelo Vicariotto, Antonio Pacifico, Pietro Storelli, Makenna Mahrer, Emanuele Venanzi, Francesca Romana Reinero

**Affiliations:** 1 Sharks Studies Center—Scientific Institute, 58024 Massa Marittima, Italy; vicariottoc@gmail.com (C.V.); storellipietro@gmail.com (P.S.); fr.reinero@gmail.com (F.R.R.); 2 Department of Physical Sciences, Earth and Environment, University of Siena, 53100 Siena, Italy; 3 Department of Life and Environmental Sciences, University of Cagliari, 09126 Cagliari, Italy; marco.pireddu.mp00@gmail.com (M.P.);; 4 Department of Life and Environmental Sciences, Polytechnic University of Marche, 60131 Ancona, Italy; damianopersia99@gmail.com; 5 Department of Economics and Law, University of Macerata, 62100 Macerata, Italy; antonio.pacifico86@gmail.com; 6 W. M. Keck Science Department, Claremont McKenna College, Claremont, CA 91711, USA; 7 Manta Diving Nosy Be, Nosy Be 207, Madagascar; info@mantadiveclub.it

**Keywords:** photo-identification, Madagascar, nursery, shark aggregation, *Carcharhinus amblyrhinchos*

## Abstract

**Simple Summary:**

Between 2012 and 2023, using non-invasive photo-identification techniques, 23 specimens of grey reef sharks were identified in the Mozambique Channel off Nosy Be, in an area called “Mokarran”. Some specimens have been re-sighted in this area for up to 1982 days. A probable aggregation and nursery area for this species may have been identified off the coast of Madagascar and, if confirmed, it will be necessary to provide for its protection.

**Abstract:**

Following preliminary underwater observations of about 1000 h carried out monthly between 2012 and 2023 (except the years 2021 and 2022), 23 specimens of grey reef sharks were spotted and photo-identified off the coast of Nosy Be in Madagascar, on an emerging reef called “Mokarran” at a depth between 15 and 30 m. Over 10 years of observations, eight specimens were re-sighted, identified with a non-invasive photo-identification technique of part of the first dorsal and the caudal fin, and one specimen was re-identified after 1982 days from the first sighting, i.e., after more than 5 years. In addition, six specimens of probably pregnant females were also identified in the same area. The population was entirely made up of females. The aggregation area could represent a new nursery site which, if confirmed after further investigations, will require greater protection.

## 1. Introduction

The grey reef shark *Carcharhinus amblyrhynchos* (Bleeker, 1856) is included in the IUCN red list as Endangered A2bcd. Relatively little is known about the biology of this species and it is estimated to have undergone a population reduction of 50–79% over the last three generations (44 years) due to exploitation and declines in habitat quality [1]. *C. amblyrhynchos* is one of Madagascar’s 135 species of sharks and rays [2]. It was described for the first time in this area in 1961 [3]. Reproduction of the grey reef shark occurs between June and July in the west coast of Madagascar, and it reaches sexual maturity in this area at around 135 cm in total length (TL), with four new pups born during each parturition with a size ranging from 50 to 67 cm [3]. To date, there is not yet comprehensive national-level legislation regulating shark fishing in Madagascar. Shark harvests in Madagascar’s waters occur both due to targeted catch and bycatch from primarily foreign-based industrial fishing companies targeting shrimp, tuna, and other pelagic species [2]. Furthermore, monitoring data about the grey reef shark population are not available for Madagascar, except for a first description of the artisanal shark fishery in the northern area [4].

Site fidelity is a common phenomenon in many species, including the whitetip reef shark *Triaenodon obesus* (Ruppel, 1837), the nurse shark *Ginglymostoma cirratum*, the blacktip reef shark *Carcharhinus melanopterus* (Quoy and Gaimard, 1824), the Caribbean grey reef shark *Carcharhinus perezi* (Poey, 1876), and the grey reef shark *C. amblyrhyncos* [5,6,7,8,9,10]. The degree of fidelity in sharks appears to vary according to life history stage, availability of resources, and area of suitable habitat [11]. Site fidelity is also common in adult reef sharks, although typically more sporadic when compared to juveniles, which might be partially explained by ontogenetic increases in the size of home ranges [12]. Fish aggregations can be random, dictated by attraction to a common resource, or real social groups between specific individuals with an organized structure and a clear purpose, such as for protection from predators or facilitating foraging [13]. In the case of elasmobranchs, the most recently accepted definition of aggregation is the co-occurrence of two or more individuals in space and time due to the deliberate use of a common driver [14]. Concerning the grey reef shark site fidelity behavior, the data available are discordant on whether they display site fidelity. Studies carried out in Australia showed limited reef fidelity and evidence of large-scale movements within northern Australian waters [11]. Conversely, site fidelity and residency of grey reef sharks on the outer slope of coral reefs in Palau, Micronesia, were detected regularly over a period of two years and nine months, and sharks displayed strong inter-annual residency with greater attendance at monitored sites during summer rather than winter months [15]. It is commonly believed that grey reef sharks display strong levels of site fidelity that persist across years, at least for some portion of the population, and reef-associated sharks generally show high residency to a single reef [10,16,17,18]. In addition, the site fidelity and the vertical movements of *C. amblyrhynchos* also vary daily depending on the phases of the moon and may be related to foraging, and sharks seasonally avoid colder waters at depth during the winter [15,17,19]. Furthermore, site fidelity is possibly linked to various aspects of the life cycle of *C. amblyrhynchos* in many populations of the species and may lead to the formation of aggregations in areas characterized by this fidelity [15].

Off Nosy Be in Madagascar, in the Mozambique Channel, we observed a grey reef shark aggregation site around the pinnacle of a coral reef at about 15–30 m depth. It is of primary importance to identify each specimen in order to monitor the population present in the aggregation site. Researchers are seeking to develop non- or minimally invasive methods to investigate elasmobranch biology and ecology [20,21], in particular for studies on threatened and protected species [11]. Photo-identification (photo-ID) offers a valid alternative to conventional tagging methods where its assumptions and practical constraints are met, in some cases improving the results of mark–recapture studies [22].

Photo-ID is a non-invasive technique and is used for several sharks species, including the white shark *Carcharodon carcharias* (Linnaeus, 1758) [23,24,25], the whale shark *Rhincodon typus* (Smith, 1828) [26,27], the ornate wobbegong shark *Orectolobus ornatus* (De Vis, 1883) [28], the zebra shark *Stegostoma fasciatum* (Hermann, 1783) [29], and the blacktip reef shark [30]. Photo-ID (both photo and video) is generally considered to be the best method for recording the appearance of natural markings or scars, and uniform composite sketches of each identified individual can be compiled as a quick reference catalogue to compare subsequent encounters [24,31]. Identifying and reporting aggregation areas for this species is a priority to promote its proper conservation, as marine protected areas provide a large benefit for the general protection and conservation of sharks [32], even if behavioral differences between sexes and across life stages of *C. amblyrhynchos* suggest that marine reserves may provide lower protection relative to more remote and isolated coral reefs [33]. The purpose of these observations was to optimize the techniques for identifying individual specimens via the use of non-invasive systems and to propose an approximate estimate of the population, sex ratio, and the presence of specimens in the target site over time (2012–2023).

## 2. Materials and Methods

### 2.1. Sampling Area

**Figure 1:**
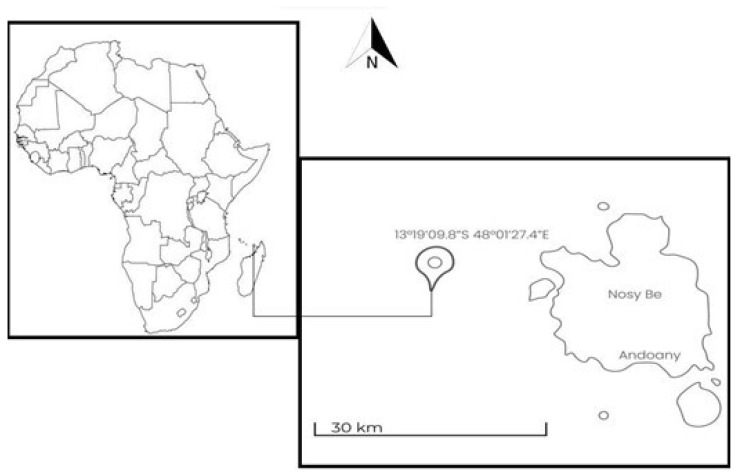
Mokarran sampling area.

Observations and data collection of the grey reef shark aggregation were carried out by the Sharks Studies Center—Scientific Institute of Massa Marittima (GR, Italy) and Manta Diving Nosy Be in Madagascar, in the waters near the island of Nosy Be (13°18′54.07″ S; 48°15′33.34″ E) in the Indian Ocean (Mozambique Channel). The average water temperature along the coast of Nosy Be is 28.1 °C, with a minimum of 25.5 °C in August and a maximum of 30.5 °C in February (SeaTemperature.org, earthobservatory.nasa.gov/global-maps/MYD28M). The photo-ID study was conducted at the site “Mokarran” (13°19′09.08″ S, 48°01′27.04″ E), at a depth between 15 and 30 m (Figure 1). The study area is characterized by a coral reef, a typical formation of tropical oceans with a high rate of biodiversity and consisting of biogenic rocks fed by the sedimentation of organisms with a calcareous skeleton. This site has a slight slope at a depth between 15 and 30 m, and then gradually flows into a sandy plateau with a depth of over 2000 m.

### 2.2. Data Collection

**Figure 2:**
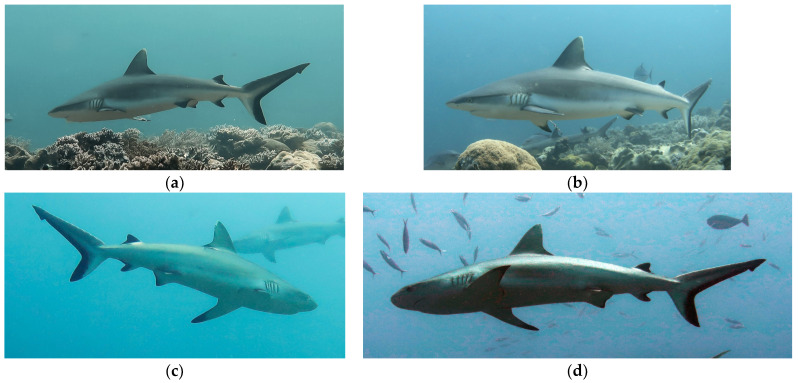
Inferior views to identify females and males (**a**–**d**).

Each year, about 10 ARA (self-contained air breathing apparatus) scuba dives were carried out monthly at the “Mokarran” site for a total of approximately 10 h of monthly underwater observations, except during the international COVID-19 pandemic years (2021–2022), and during rainy periods in January and February. Across 10 years of observations, approximately 1000 h of scuba diving were undertaken between 2012 and 2023, always in the morning between 9:00 a.m. and 2:00 p.m. Diving activities were carried out between 15 and 30 m depth. We used several underwater cameras for filming to identify individual specimens and their behavior. For the underwater videos and photos, the Canon G16, iPhone 6S plus with Easydive diving case, Leo 3 smart, and VolkanoX Cam were used. Accepted images had to have at least a minimum of 1 MB of data, 96 DPI resolution, and dimensions of 2160 × 1441 pixels. The shark size at sexual maturity, measured in total length (TL), was estimated when grey reef sharks passed in front of a diver as a size reference of about 180 cm in TL to the nearest 0.2 m. Male grey reef sharks mature at 110–145 cm TL, while females at 120–142 cm TL [34,35]. The females were recorded if a lack of claspers was verified and their pelvic fin area was filmed or photographed (Figure 2a–d).

### 2.3. Photo Collection Procedure and Specimen Photo-Identification Protocol

**Figure 3:**
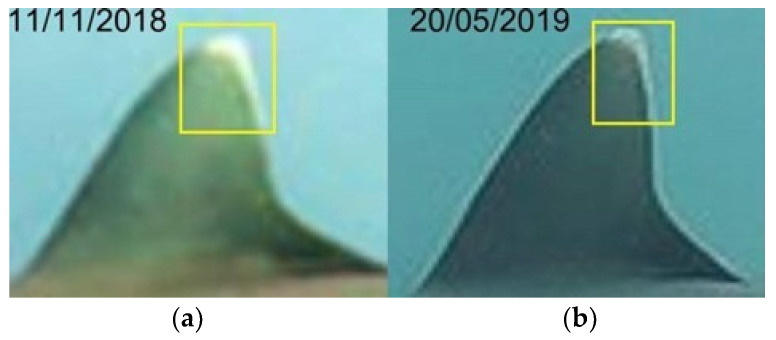
Identification area of the first dorsal fin in the same grey reef shark over time, years 2018 (**a**), 2019 (**b**) (resighting 6).

**Figure 4:**
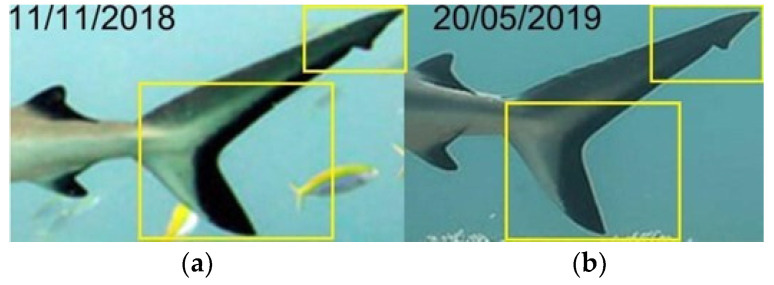
Identification area, in the dorsal fin, choosed to identify grey reef sharks over time, years 2018 (**a**), 2019 (**b**) (resighting 6).

**Figure 5:**
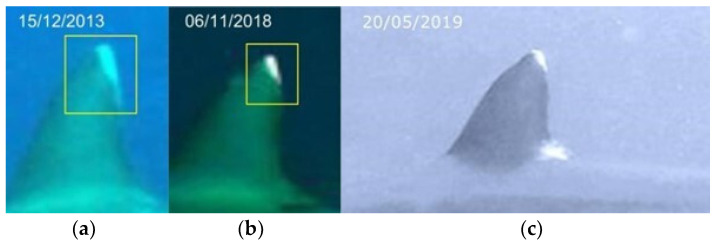
Identification area, in the dorsal fin, choosed to identify grey reef sharks over time, years 2013 (**a**), 2018 (**b**) and 2019 (**c**) (resighting 1).

**Figure 6:**
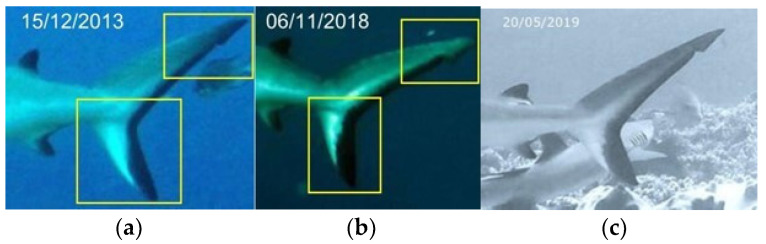
Identification area of the caudal fin in the same grey reef shark over time, years 2013 (**a**), 2018 (**b**) and 2019 (**c**) (resighting 1).

**Figure 7:**
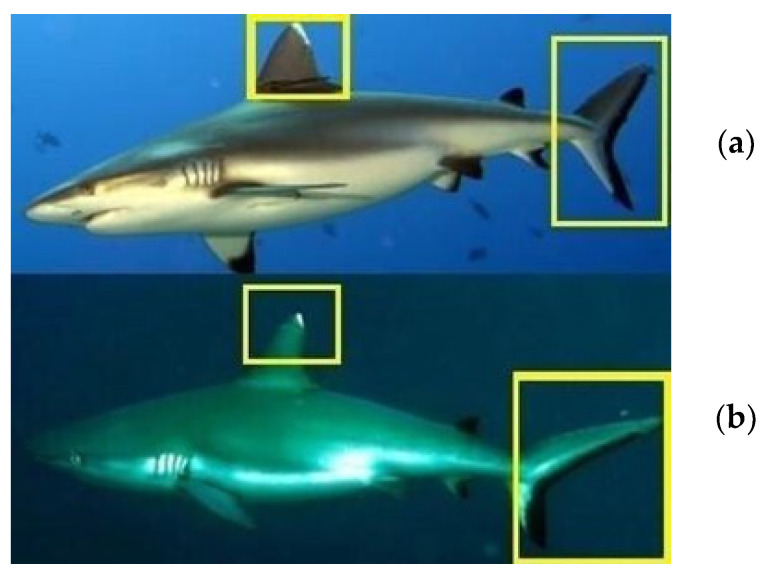
Comparison areas, in the dorsal fins and caudal fins, choosed to identify different grey reef sharks over time (**a**,**b**).

The identification of grey reef sharks was carried out with a non-invasive identification technique that analyzes and compares the shape and color in the first dorsal and caudal fin tips, including scars, bite marks, fin morphology, and deformities useful in the absence of intrinsic patterns [30,36,37,38]. The photo-ID of *C. amblyrhynchos* was conducted by studying and comparing the intraspecific differences present in the first dorsal and caudal fin and some examples are shown in Figure 3a,b, Figure 4a,b, Figure 5a–c, Figure 6a–c and Figure 7a,b. However, it is important to underline that the identification photographs did not include all the animals sighted due to the cryptic behavior of grey reef sharks, which rarely approached divers. Both distance and water turbidity critically reduced the resolution of some photographs, making photo-identification impossible. Furthermore, it was not possible to photograph both sides of every specimen, reducing the researcher’s ability to assess unique marking characteristics.

The photo-ID protocol was divided into the following phases:

- Videos were collected by each member of the team and divided by specimen and temporal sequence;
- Stills were taken from the videos in which the fundamental features for photo-ID were clearly visible;
- New specimens were compared with the ones already present in the database to verify that they were not a resighting, comparisons were performed using Excel;
- Photos of each shark were loaded into the Excel sheet relating to the year of sighting and any related data were loaded into the appropriate table. Image processing included removing the background and highlighting the first dorsal, especially the area with the white spot and caudal fin black spot. Additional excess parts were removed using the “mark areas to keep” or “mark areas to remove” operations.

## 3. Results

**Figure 8:**
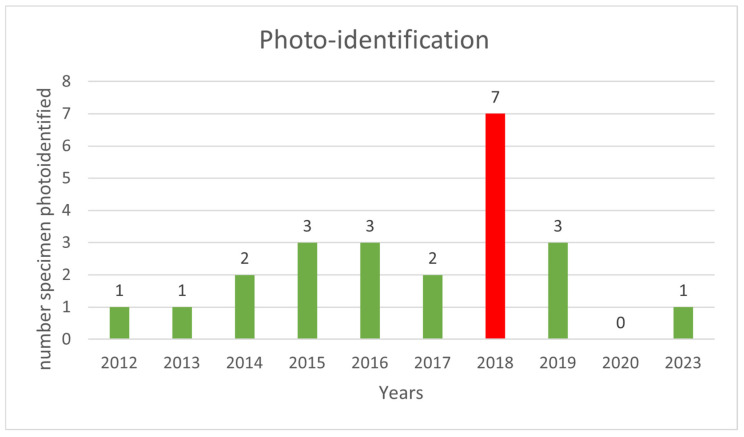
Yearly frequency of photo-identified specimens of *C. amblyrhynchos* from 2012 to 2023 (except for the years 2021 and 2022). In 2018 (red column), the highest number of grey reef sharks (n = 7) photo-identified was observed.

**Figure 9:**
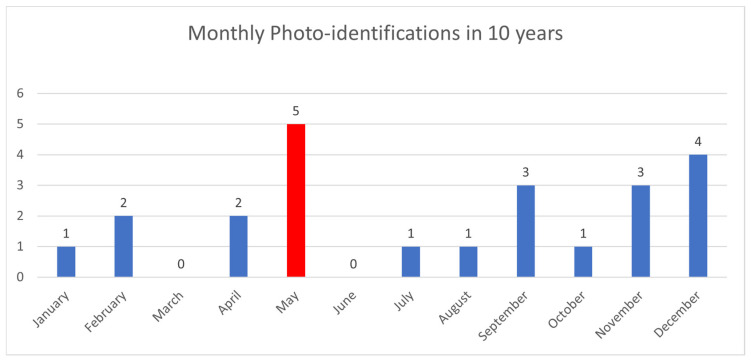
Monthly frequency of photo-identified specimens of *C. amblyrhynchos* in 10 years. May (red column) was the month with the highest peak sightings (n = 5).

**Figure 10:**
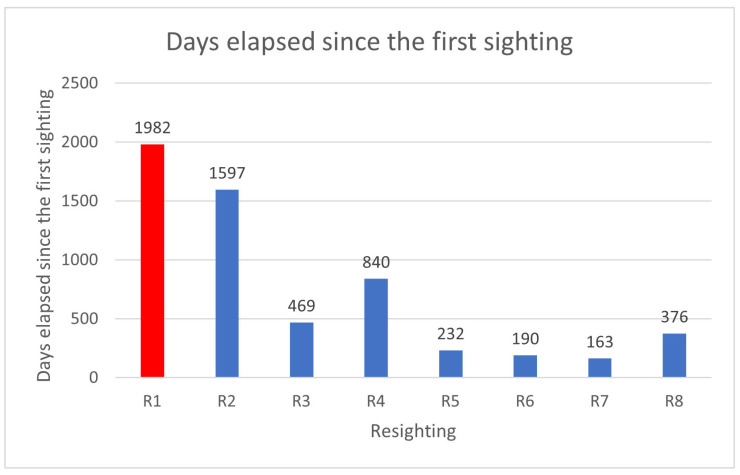
Days elapsed after the first sighting of *C. amblyrhynchos* individuals classified as resightings (R). Resightings can only be defined as such if the sightings occur from one year to another. R1 (red column) was an individual re-sighted 1982 days after the first identification (first identification occurred on 6 November 2018 and the second one five years later on 15 December 2023).

**Figure 11:**
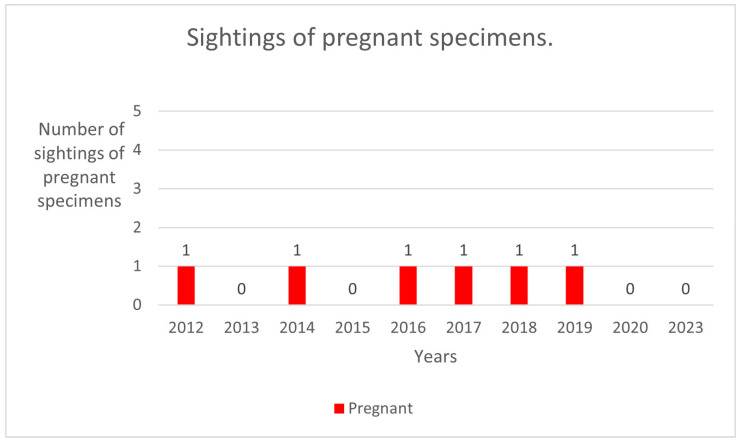
Sightings of likely pregnant grey reef sharks.

In the sampling area “Mokarran,” in the 10 years between 2012 and 2023 (except the years 2021 and 2022), a total of about 1000 h of underwater observations by scuba diving were completed. Of the 54 sightings documented during this period, only 23 *C. amblyrhynchos* specimens were identified (Figure 8, Table 1), and both sides were photographed for only 3 specimens; 16 specimens were captured from the left side and 4 were captured from the right side. Specimens observed and identified were all females indicating a sex ratio of 1:0 and shark size ranged between 50 cm and 180 cm in TL. The yearly identification peak was observed in 2018 and 2019, while monthly peak sightings were observed in May and November (Figure 9). A total of eight resightings were observed, with the longest being recorded more than 5 years later after 1982 days (Figure 6, Figure 7 and Figure 10; Table 2). In total, six likely pregnant females were photo-identified (Figure 11) and specimen number 2, sighted from 2013 to 2019, was probably pregnant in 2018 (Figure 9). A few juveniles around 50 cm in TL were sighted during the 10 years of observations (personal comment E. Venanzi). To have a better chance of encountering sharks, the opposite current seems to favor the ascent and aggregation of *C. amblyrhynchos* toward the upper edge of the coral reef, thus favoring close observation of the animals (personal comment E. Venanzi) [39].

## 4. Discussion

Shark aggregations can occur for various reasons under the most recent definition of the term, as previously explained [14]. The fidelity to the site found in various species of sharks—and in particular in grey reef sharks—involves an aggregation activity, in a specific area, for various purposes, including but not limited to hunting, reproduction, and nursery activities [15,40]. Off the coast of Nosy Be in Madagascar, in the Mozambique Channel, on a coral drop-off at a depth between 15 and 30 m, we observed the presence of 23 specimens of grey reef sharks over 10 years, but since their sighting is linked to their patrolling behavior around the emerging reef, it is difficult to be able to film and photograph both sides in order to confirm a unique identification of each individual. Consequently, the number of 23 specimens can be interpreted within a range of 19 to 23 possible different individuals actually identified out of the 54 total sightings. The development of a non-invasive identification technique based on the photo-ID of a particular area of the first dorsal and the caudal fin gave us the possibility to re-observe some of the same specimens, and one in particular (R1), 5 years after the first sighting. The number of identified resightings was low. Only 8 individuals were re-sighted out of approximately 19–23 different specimens, which does not compellingly suggest the presence of site fidelity behavior in this area. The nursery area hypothesis can be defined based on three primary criteria for newborn or young-of-the-year (YOY) individuals (i.e., individuals < 1 year old): (1) sharks are more commonly encountered in the area than in other areas, i.e., density in the area is greater than the mean density over all areas; (2) sharks have a tendency to remain or return for extended periods (weeks or months), i.e., site fidelity is greater than the mean site fidelity for all areas; (3) the area or habitat is repeatedly used across years, whereas others are not [41]. Despite the well-documented growth and reproduction biology of *C. amblyrhynchos*, little understanding exists of the distribution and or physical description of what constitutes optimal nursery conditions for this species [42]. The yearly presence of pregnant females and several specimens measuring around 50 cm (personal comment E. Venanzi) could suggest the possibility of the site being a nursery area. Additionally, given that the dispersal abilities of *C. amblyrhynchos* are similar throughout the Indo-Pacific and are independent of the availability of coral reefs [43], it is important to understand where they come from and where they go to improve the photo-ID technique in combination with genetic sampling. The population peaks observed mainly in May could be linked to the reproductive period indicated to be between June and July in Madagascar by Furmanoir (1961), even though pregnant females were observed in August and November. The total absence of males could go in the direction of confirming a nursery area with sexual segregation [44,45].

Reef sharks can alter ecologically important behaviors of mesopredators. It is likely that reef sharks represent a competitor rather than simply a predator and have the capacity to play an important regulatory role for mesopredator populations in coral reefs via interference competition [46]; in fact, defining the roles of species within marine ecosystems is complicated due to a lack of observed interactions amongst species [47]. To clarify the roles of reef sharks, ref. [18] classified sharks into categories based on body size and trophic level. This classification resulted in most requiem shark species (e.g., grey reef, blacktip reef, whitetip reef, and Caribbean reef sharks) being classified as mesopredators [46]. Recent studies have supported this classification and shown that requiem reef sharks occupy similar trophic levels and isotopic niche space to large-bodied teleost predators [48,49,50]. This seemingly high level of trophic redundancy could explain the limited evidence for shark-induced trophic cascades in most coral reef studies [48,49], as these species are likely acting as mesopredators rather than apex predators [18,50,51]. In addition, understanding and defining the role that the wider range of shark species play in coral reef ecosystems and the level of interaction between resident and non-resident species remains a crucial topic given the current declines in top predators [47]. Thus, understanding these topics in the next project steps is important in this area, especially if the presence of a nursery area could be confirmed in the future.

## 5. Conclusions

From the data collected between 2012 and 2023, we can hypothesize as follows: (i) given that researchers observed exclusively female specimens, of which six were apparently pregnant, it is possible to suggest that the “Mokarran” site is a nursery area; (ii) the best month to carry out monitoring activities on the population of *C. amblyrhynchos* seems to be May, perhaps linked to a parturition period; and (iii) the resighting of eight individuals in the site in the following years, after a minimum of 163 days to a maximum of 1982 days, indicates—even if it does not definitively confirm—that the identification method can be further applied to be validated in the long term by genetic sampling. The next steps of this project will be aimed at confirming the total number of identified specimens, including pregnant females and YOY resightings. Understanding the role played by this species in the identified area can be used to suggest best methods and purposes for the conservation of this new aggregation site and provide a deeper knowledge of its dispersal in other areas. Photo-ID should be further validated by comparing photographic data with genetic samples of individual specimens to confirm the uniqueness of each specimen, integrating it with optimization of the technique for measuring sizes via laser photogrammetry and the use of the BRUVS (baited remote underwater video system), for closer observations. The recent shark sanctuary put in place in the northeast of Madagascar could provide a template for the growth of nearshore shark fisheries management in Madagascar through the established network of >65 locally managed marine areas (LMMAs) covering > 11,000 km^2^ [52], and this new area, “Mokarran”, could be included in a proposal for a new northwest Madagascar shark sanctuary. In particular, if the nursery area is confirmed, it will be important to suggest the establishment of an ISRA (Important Sharks and Ray Area).

## Figures and Tables

**Table 1:** Distribution of sightings and identifications from 2012 to 2023 (except the years 2021 and 2022).

Year	Sightings	Identifications
2012	1	1
2013	1	1
2014	2	2
2015	5	3
2016	6	3
2017	5	2
2018	19	7
2019	13	3
2020	1	0
2023	1	1
Total	54	23

**Table 2:** Photo-ID dates of individuals classified as resightings (R).

N. Photo-ID	Date First Photo-ID	Date Second Photo-ID	Date Third Photo-ID
N.2-R1	15 December 2013	6 November 2018 Pregnant	20 May 2019
N.5-R2	2 January 2015	4 November 2018	18 May 2019 Pregnant
N.8-R3	26 May 2016 Pregnant	7 September 2017	
N.9-R4	17 July 2016	21 August 2017 Pregnant	4 November 2018
N.13-R5	28 September 2018	18 May 2019	
N.16-R6	11 November 2018	20 May 2019	
N.17-R7	6 December 2018	18 May 2019	
N.20-R8	16 May 2019	26 June 2020	

## Data Availability

Data will be available after publication on Researchgate after request to the authors.

## References

[1] Simpfendorfer C., Fahmi A.B.A., Utzurrum J.A.T., Seyha L., Maung A., Bineesh K.K., Yuneni R.R., Sianipar A., Haque A.B., Tanay D. (2020). *Carcharhinus amblyrhynchos*. The IUCN Red List of Threatened Species. http://www.iucnredlist.org/species/pdf/173433550.

[2] Cripps G., Harris A., Humber F., Harding S., Thomas T. (2015). A Preliminary Value Chain Analysis of Shark Fisheries in Madagascar. Programme for the Implementation of a Regional Fisheries Strategy for the Eastern and Southern Africa Indian Ocean Region.

[3] Fourmanoir P. (1961). Requins de la cote ouest de Madagascar. Mémoires de l’Institut Scientifique de Madagascar. Série F. Océanogr..

[4] Robinson L., Sauer W. (2013). A first description of the artisanal shark fishery in northern Madagascar: Implications for management. Afr. J. Mar. Sci..

[5] Nelson D.R., Johnson R.H. (1980). Behavior of the reef sharks of Rangiroa, French Polynesia. Natl. Geogr. Soc. Res. Rep..

[6] Castro A.L., Rosa R.S. (2005). Use of natural marks on population estimates of the nurse shark, *Ginglymostoma cirratum*, at Atol das Rocas Biological Reserve, Brazil. Environ. Biol. Fishes.

[7] Chapman D.D., Pikitch E.K., Babcock E., Shivji M.S. (2005). Marine reserve design and evaluation using automated acoustic telemetry: A case-study involving coral reef-associated sharks in the Mesoamerican Caribbean. Mar. Technol. Soc. J..

[8] Papastamatiou Y.P., Lowe C.G., Caselle J.E., Friedlander A.M. (2009). Scale dependent effects of habitat on movements and path structure of reef sharks at a predator-dominated atoll. Ecology.

[9] Fitzpatrick R., Abrantes K.G., Seymour J., Barnett A. (2011). Variation in depth of whitetip reef sharks: Does provisioning ecotourism change their behaviour?. Coral Reefs.

[10] Barnett A., Abrantes K.G., Seymour J., Fitzpatrick R. (2012). Residency and Spatial Use by Reef Sharks of an Isolated Seamount and Its Implications for Conservation. PLoS ONE.

[11] Heupel M.R., Simpfendorfer C.A. (2010). Science or slaughter: Need for lethal sampling of sharks. Conserv. Biol..

[12] Speed C.W., Field I.C., Meekan M.G., Bradshaw C.J.A. (2010). Complexities of coastal shark movements and their implications for management. Mar. Ecol. Prog. Ser..

[13] Krause J., Hensor E.M.A., Ruxton G.D., Hart P.J.B., Reynolds J.D. (2002). Fish as prey. Handbook of Fish Biology and Fisheries. Fish Biology.

[14] McInturf A.G., Bowman J., Schulte J.M., Newton K.C., Vigil B., Honig M., Pelletier S., Cox N., Lester O., Cantor M. (2023). A unified paradigm for defining elasmobranch aggregations. ICES J. Mar. Sci..

[15] Vianna G.M.S., Meekan M.G., Meeuwig J.J., Speed C.W. (2013). Environmental Influences on Patterns of Vertical Movement and Site Fidelity of Grey Reef Sharks (*Carcharhinus amblyrhynchos*) at Aggregation Sites. PLoS ONE.

[16] Field I.C., Meekan M.G., Speed C.W., White W., Bradshaw C. (2011). Quantifying movement patterns for shark conservation at remote coral atolls in the Indian Ocean. Coral Reefs.

[17] Bond M.E., Babcock E.A., Pikitch E.K., Abercrombie D.L., Lamb N.F., Chapman D.D. (2012). Reef Sharks Exhibit Site-Fidelity and Higher Relative Abundance in Marine Reserves on the Mesoamerican Barrier Reef. PLoS ONE.

[18] Heupel M.R., Simpfendorfer C.A. (2014). Importance of environmental and biological drivers in the presence and space use of a reef-associated shark. Mar. Ecol. Prog. Ser..

[19] McKibben J.N., Nelson D.R. (1986). Patterns of movement and grouping of grey reef sharks, *Carcharhinus amblyrhynchos*, at Enewetak, Marshall Islands. Bull. Mar. Sci..

[20] Awruch C.A., Frusher S.D., Pankhurst N.W., Stevens J.D. (2008). Non-lethal assessment of reproductive characteristics for management and conservation of sharks. Mar. Ecol. Prog. Ser..

[21] Barnett A., Redd K.S., Frusher S.D., Stevens J.D., Semmens J.M. (2010). Non-lethal method to obtain stomach samples from a large marine predator and the use of DNA analysis to improve dietary information. J. Exp. Mar. Biol. Ecol..

[22] Rowat D., Speed C.W., Meekan M.G., Gore M. (2009). Population abundance and apparent survival of the Vulnerable whale shark, *Rhincodon typus*, in the Seychelles aggregation. Oryx.

[23] Sperone E., Micarelli P., Andreotti S., Brandmayr P., Bernabò I., Brunelli E., Tripepi S. (2012). Surface behaviour of bait-attracted white sharks at Dyer Island (South Africa). Mar. Biol. Res..

[24] Micarelli P., Sperone E., Pecchia JGiglio G., Mele F., Scuderi A., Romano C., Vespaziani L. (2015). Dorsal fin photoidentification: Tool for long term studies of White shark (*Carcharodon carcharias*) behaviour. Biol. Mar. Mediterr..

[25] Micarelli P., Bonsignori D., Compagno L.J.V., Pacifico A., Romano C., Reinero F.R. (2021). Analysis of sightings of white sharks in Gansbaai (South Africa). Eur. Zool. J..

[26] Arzoumanian Z., Holmberg J., Norman B. (2005). An astronomical pattern-matching algorithm for computer-aided identification of whale sharks, Rhincodon typus. J. Appl. Ecol..

[27] Meekan M.G., Bradshaw C.J.A., Press M., McLean C., Richards A., Quasnichka S., Taylor J.G. (2006). Population size and structure of whale sharks (*Rhincodon typus*) at Ningaloo Reef, Western Australia. Mar. Ecol. Prog. Ser..

[28] Carraro R., Gladstone W. (2006). Habitat preferences and site fidelity of the ornate wobbegong shark (*Orectolobus ornatus*) on rocky reefs of New South Wales. Pac. Sci..

[29] Dudgeon C.L., Noad M.J., Lanyon J.M. (2008). Abundance and demography of a seasonal aggregation of zebra sharks *Stegostoma fasciatum*. Mar. Ecol. Prog. Ser..

[30] Mourier J., Vercelloni J., Planes S. (2012). Evidence of social communities in a spatially structured network of a free-ranging shark species. Anim. Behav..

[31] Marshall A.D., Pierce S.J. (2012). The use and abuse of photographic identification in sharks and rays. J. Fish Biol..

[32] Martín G., Espinoza M., Heupel M., Simpfendorfer C.A. (2020). Estimating marine protected area network benefits for reef sharks. J. Appl. Ecol..

[33] Espinoza M., Heupel M.R., Tobin A.J., Simpfendorfer C.A. (2015). Residency patterns and movements of grey reef sharks (*Carcharhinus amblyrhynchos*) in semi-isolated coral reef habitats. Mar. Biol..

[34] Wetherbee B.M., Crow G.L., Lowe C.G. (1997). Distribution, reproduction and diet of the Grey Reef Shark *Carcharhinus amblyrhynchos* in Hawaii. Mar. Ecol. Prog. Ser..

[35] Ebert D.A., Fowler S.L., Compagno L.J.V. (2013). Sharks of the World: A Fully Illustrated Guide.

[36] Klimley A.P., Anderson S.D., Klimley A.P., Ainley D.G. (1996). Residency patterns of white sharks at the South Farallon Islands, California. Great White Sharks: The Biology of Carcharodon carcharias.

[37] Sims D.W., Speedy C.D., Fox A.M. (2000). Movements and growth of a female basking shark re-sighted after a three-year period. J. Mar. Biol. Assoc..

[38] Anderson S.D., Chapple T.K., Jorgensen S.J., Klimley A.P., Block B.A. (2011). Long-term individual identification and site fidelity of white sharks, *Carcharodon carcharias*, off California using dorsal fins. Mar. Biol..

[39] Hussey N.E., Stroh N., Klaus R., Chekchak T., Kessel S.T. (2013). SCUBA diver observations and placard tags to monitor grey reef sharks, Carcharhinus amblyrhynchos, at Sha’ab Rumi, The Sudan: Assessment and future directions. J. Mar. Biol. Assoc..

[40] Mourier J., Maynard J., Parravicini V., Ballesta L., Clua E., Domeier M.L., Planes S. (2016). Extreme Inverted Trophic Pyramid of Reef Sharks Supported by Spawning Groupers. Curr. Biol..

[41] Heupel M.R., Carlson J.K., Simpfendorfer C.A. (2007). Shark nursery areas: Concepts, definition, characterization and assumptions. Mar. Ecol. Prog. Ser..

[42] Orr M. (2019). Potential Grey Reef Shark (*Carcharhinus amblyrhynchos*) Nursery on Seamounts Southwest of Guam. Micronesica.

[43] Lesturgie P., Braun C.D., Clua E., Mourier J., Thorrold S.R., Vignaud T., Planes S., Mona S. (2023). Like a rolling stone: Colonization and migration dynamics of the grey reef shark (*Carcharhinus amblyrhynchos*). Ecol. Evol..

[44] Klimley A.P. (1987). The determinants of sexual segregation in the scalloped hammerhead shark, *Sphyrna lewini*. Environ. Biol. Fishes.

[45] Mourier J., Mills S.C., Planes S. (2013). Population structure, spatial distribution and life-history traits of blacktip reef sharks *Carcharhinus melanopterus*. J. Fish Biol..

[46] Lester E. (2021). Investigating the Influence of Reef Sharks on the Behavior of Mesopredatorsin Dynamic Coral Reef Seascapes. Ph.D. Thesis.

[47] Heupel M.R., Simpfendorfer C.A., Olsen E.M., Moland E. (2012). Consistent movement traits indicative of innate behavior in neonate sharks. J. Exp. Mar. Biol. Ecol..

[48] Frisch A.J., Ireland M., Rizzari J.R., Lomstedt O.M., Magnenat K.A., Mirbach C.E., Hobbs J.P.A. (2016). Reassessing the trophic role of reef sharks as apex predators on coral reefs. Coral Reefs.

[49] Casey J.M., Baird A.H., Brandl S.J., Hoogenboom M.O., Rizzari J.R., Frisch A.J., Mirbach C.E., Connolly S.R. (2017). A test of trophic cascade theory: Fish and benthic assemblages across a predator density gradient on coral reefs. Oecologia.

[50] Bond M.E., Valentin-Albanese J., Babcock E.A., Hussey N.E., Heithaus M.R., Chapman D.D. (2018). The trophic ecology of Caribbean reef sharks (*Carcharhinus perezi*) relative to other large teleost predators on an isolated coral atoll. Mar. Biol..

[51] Roff G., Doropoulos C., Rogers A., Bozec Y.M., Krueck N.C., Aurellado E., Priest M., Birrel C., Mumby P.J. (2016). The ecological role of sharks on coral reefs. Trends Ecol. Evol..

[52] Mihari (2016). MIAHRI−Madagascar’s Locally Managed Marine Area Network. http://mihari-network.org.

